# (4-Chloro­phen­yl)(2,7-dimeth­oxy-8-nitro­naphthalen-1-yl)methanone

**DOI:** 10.1107/S1600536810051998

**Published:** 2010-12-18

**Authors:** Takahiro Nishijima, Atsushi Nagasawa, Teruhisa Takada, Akiko Okamoto, Noriyuki Yonezawa

**Affiliations:** a Department of Organic and Polymer Materials Chemistry, Tokyo University of Agriculture & Technology, 2-24-16 Naka-machi, Koganei, Tokyo 184-8588, Japan

## Abstract

In the title compound, C_19_H_14_ClNO_5_, the aroyl group is attached to the naphthalene ring system with a non-coplanar configuration. The dihedral angle between naphthalene ring system and benzene ring is 70.62 (6)°. The nitro group is oriented in parallel with the adjacent carbonyl plane. The torsion angle of the carbonyl group and naphthalene ring is 54.68 (19)° (C—C—C—O), and that of nitro group and naphthalene ring is 54.26 (18)° (O—N—C—C). In the crystal, π–π inter­actions between naphthalene systems [centroid–centroid distances = 3.5633 (9), 3,5634 (9), and 3.9758(9) Å], C—H⋯O hydrogen bonds, inter­molecular N—O⋯Cl inter­actions [2.9937 (12) Å] and C—H⋯π contacts are observed.

## Related literature

For electrophilic aromatic substitution of naphthalene derivatives giving aryl naphthyl ketone compounds, see: Okamoto & Yonezawa (2009 ▶). For related structures, see: Kato *et al.* (2010 ▶); Mitsui *et al.* (2008 ▶, 2010 ▶); Nishijima *et al.* (2010 ▶); Watanabe *et al.* (2010 ▶).
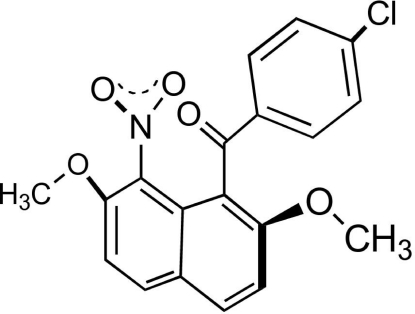


## Experimental

### 

#### Crystal data

C_19_H_14_ClNO_5_
*M* *_r_* = 371.76
Monoclinic, 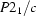

*a* = 8.57511 (16) Å
*b* = 14.0424 (3) Å
*c* = 14.0842 (3) Å
β = 100.206 (1)°
*V* = 1669.12 (5) Å^3^
*Z* = 4
Cu *K*α radiation
μ = 2.31 mm^−1^
*T* = 193 K
0.60 × 0.40 × 0.30 mm

#### Data collection

Rigaku R-AXIS RAPID diffractometer
Absorption correction: numerical (*NUMABS*; Higashi, 1999 ▶) *T*_min_ = 0.294, *T*_max_ = 0.544
29408 measured reflections
3058 independent reflections
2793 reflections with *I* > 2σ(*I*)
*R*_int_ = 0.028

#### Refinement

*R*[*F* ^2^ > 2σ(*F* ^2^)] = 0.033
*wR*(*F* ^2^) = 0.093
*S* = 1.03
3058 reflections
238 parameters
H-atom parameters constrained
Δρ_max_ = 0.21 e Å^−3^
Δρ_min_ = −0.19 e Å^−3^

### 

Data collection: *PROCESS-AUTO* (Rigaku, 1998 ▶); cell refinement: *PROCESS-AUTO*; data reduction: *CrystalStructure* (Rigaku/MSC, 2004 ▶); program(s) used to solve structure: *SIR2004* (Burla *et al.*, 2005 ▶); program(s) used to refine structure: *SHELXL97* (Sheldrick, 2008 ▶); molecular graphics: *ORTEP* (Burnett & Johnson, 1996 ▶); software used to prepare material for publication: *CrystalStructure*.

## Supplementary Material

Crystal structure: contains datablocks I, global. DOI: 10.1107/S1600536810051998/om2387sup1.cif

Structure factors: contains datablocks I. DOI: 10.1107/S1600536810051998/om2387Isup2.hkl

Additional supplementary materials:  crystallographic information; 3D view; checkCIF report

## Figures and Tables

**Table 1:** Hydrogen-bond geometry (Å, °) *Cg*1 is the centroid of the C1–C4/C9/C10 ring.

*D*—H⋯*A*	*D*—H	H⋯*A*	*D*⋯*A*	*D*—H⋯*A*
C13—H13⋯O5^i^	0.93	2.44	3.137 (2)	132
C18—H18*C*⋯O3^i^	0.96	2.51	3.449 (2)	167
C19—H19*B*⋯*Cg*1^ii^	0.96	2.81	3.5845 (19)	139

Symmetry codes: (i) 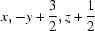; (ii) 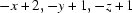.

**Fig. 1.**
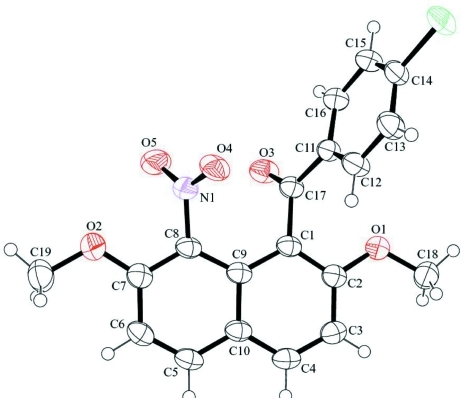
Molecular structure of the title compound with displacement ellipsoids at the 50% probability level.

**Fig. 2.**
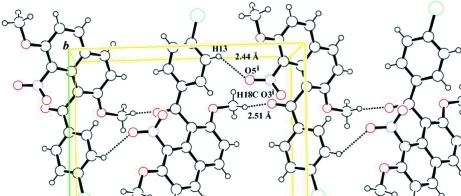
A partial crystal packing diagram of the title compound, viewed down the b axis. The intermolecular C—H···O hydrogen bonds are shown as dashed lines.

**Fig. 3.**
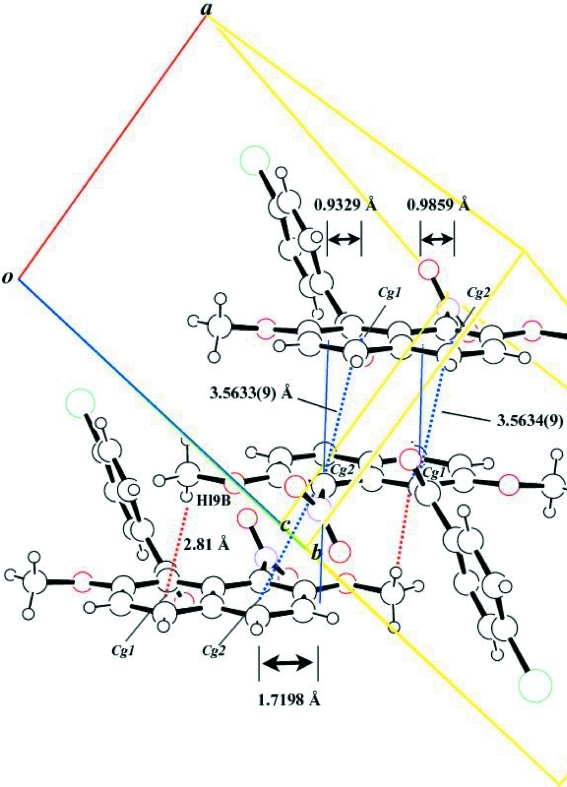
Side view of the C—H···π interactions (red dashed lines) and the π—π interactions (blue dashed lines).

**Fig. 4.**
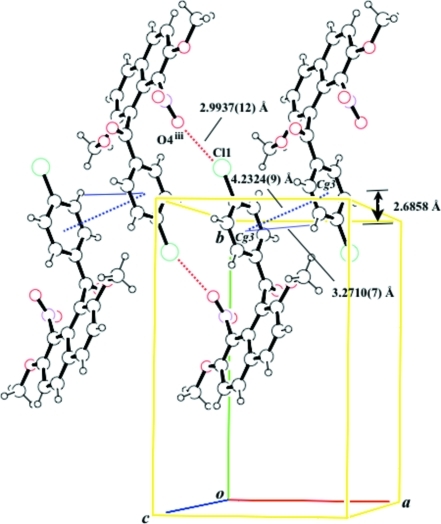
Side view of the O···Cl interactions (red dashed lines) and the π—π interactions (blue dashed lines).

## supplementary crystallographic information

### Comment

In the course of our study on electrophilic aromatic aroylation of 2,7-dimethoxynaphthalene, *peri*-aroylnaphthalene compounds have proven to be formed regioselectively with aid of suitable acidic mediators (Okamoto & Yonezawa, 2009). Recently, we have reported the crystal structures of several 1,8-diaroylated naphthalene homologues exemplified by 1,8-bis(4-aminobenzoyl)-2,7-dimethoxynaphthalene (Nishijima *et al*., 2010). The aromatic rings in these molecules are arranged in a non-coplanar alignment to each other. Furthermore, we have also clarified the crystal structures of 1-monoaroylated naphthalene compounds. They have essentially the same non-coplanar structure with the 1,8-diaroylated naphthalene, *e.g.*, 1-(4-chlorobenzoyl)-2,7-dimethoxynaphthalene (Mitsui *et al.*, 2008), 2,7-dimethoxy-1-(4-nitrobenzoyl)naphthalene (Watanabe *et al.*, 2010) and (2,7-dimethoxynaphthalen-1-yl)(phenyl)methanone (Kato, *et al.*, 2010). In the course of this work, we have revealed the crystal structure of 1-monoaroylnaphthalene compounds having a substituent, other than aroyl group, at the 8-position such as (8-bromo-2,7-dimethoxy-1-naphthyl)(4-chlorophenyl)methanone (Mitsui, Nagasawa, Watanabe *et al.* 2010). The aroyl group and naphthalene ring in these molecules have similar configuration to 1,8-diaroylated naphthalene. As a part of our continuous study on the molecular structures of these kinds of homologous molecules, the crystal structure of title compound, a 1-chlorobenzoylated naphthalene bearing nitro group at the 8-position, is discussed in this report.

An *ORTEP* (Burnett & Johnson, 1996) plot of the title compound is displayed in Fig. 1. In the molecule, the dihedral angle between the benzene ring (C11—C16) and the naphthalene ring (C1—C10) is 70.62 (6)°. The nitro group are also twisted away from the naphthalene ring system, then the nitro group and the carbonyl group are arranged almost in parallel. The dihedral angle between the ketonic C=O plane (O3/C1/C11/C17) and naphthalene ring (C1—C10) is 60.33 (7)° [C9—C1—C17—O3 torsion angle is 54.68 (19)°] and between the nitro plane (O4/O5/N1/C8) and naphthalene ring (C1—C10) is 57.34 (8)° [O4—N1—C8—O9 torsion angle is 54.26 (18)°]. On the other hand, the carbonyl group and the 4-chlorophenyl one have almost coplanar configuration [C16—C11—C17—O3 torsion angle is 10.5 (2)°], but the torsion angle is larger than those of another homologous compounds, *i.e.*, the torsion angles of 1-(4-chlorobenzoyl)-2,7-dimethoxynaphthalene and (8-bromo-2,7-dimethoxy-1-naphthyl)(4-chlorophenyl)methanone are -4.4 (2)° and -3.6 (4)°, respectively.

The molecular packing of the title compound is mainly stabilized by weak intermolecular hydrogen bonds and van der Waals interactions. The 4-chlorophenyl groups interact with the nitro groups [C13—H13···O5 is 2.44 Å; (i) *x*, 3/2 - *y*, 1/2 + *z*] of adjacent molecules along the *c* axis (Fig. 2). Interaction between the methoxy groups and the carbonyl groups [C18—H18C···O3 = 2.51 Å; (i) *x*, 3/2 - *y*, 1/2 + *z*] form naphthalene ring systems into zigzag arrangement (Fig. 2). On the other hand, C—H···π interaction and π–π interactions deposit layer upon layer of naphthalene rings [C1/C2/C3/C4/C9/C10 ring with centroid Cg1 and C5-C10 ring with centroid Cg2 (Fig. 3). Furthermore, O4 and C11 interact with each other [O4···Cl1 = 2.9937 (12) Å; (iii) 2 - *x*, -*y*, 1-*z*] (Fig. 4).

### Experimental

To a solution of 1-(4-chlorobenzoyl)-2,7-dimethoxynaphthalene (653.4 mg, 2.0 mmol) in methylene chloride (10 ml), aqueous 61% nitric acid (2.0 ml) was dropped by portions at 273 K. After the reaction mixture was stirred at 273 K for 2 h, it was poured into ice-cold water (25 ml). The aqueous solution was extracted with CHCl_3_(15 ml × 3). The combined extracts were washed with water followed by washing with brine. The organic layers thus obtained were dried over anhydrous MgSO_4_. The solvent was removed under reduced pressure to give cake. The crude product was purified by reprecipitation (good solvent:CHCl_3_, poor solvent:hexane) and column chromatography (silica gel, CHCl_3_) (isolated yield 448.8 mg, 60%). Single crystals suitable for X-ray diffraction analysis were obtained by crystallization from acetone as yellow plates.

Spectral data: ^1^H NMR δ (300 MHz, CDCl_3_); 3.73 (3*H*, s), 3.97 (3*H*, s), 7.22 (1*H*, d, *J* = 9.3 Hz), 7.24 (1*H*, d, *J* = 8.6 Hz), 7.38 (2*H*, d, *J* = 8.9 Hz), 7.82 (2*H*, d, *J* = 8.6 Hz), 7.95 (1*H*, d, *J* = 9.3 Hz), 7.96 (1*H*, d, *J* = 8.9 Hz). ^13^C NMR δ (75 MHz, CDCl_3_); 56.48, 57.22, 111.26, 111.86, 118.02, 124.16, 124.59, 128.64, 130.51, 132.59, 133.24, 134.39, 136.79, 139.17, 151.61, 157.76, 193.23. IR (KBr); 1655 (C=O), 1623 (Ar), 1527 (Ar), 1372 (NO_2_), 1275 (O—Me) cm^-1^. HRMS (*m/z*); [*M* + H]^+^ Calcd for C_19_H_15_ClNO_5_, 272.0639; found, 272.0601. m.p. = 483.1–486.4 K.

### Refinement

All H atoms were found in a difference map and were subsequently refined as riding atoms, with C—H = 0.93 (aromatic) Å and C—H = 0.96 (methyl) Å, and with *U*_iso_(H) = 1.2*U*_eq_(C).

### Crystal data

**Table:**

C_19_H_14_ClNO_5_	*F*(000) = 768
*M**_r_* = 371.76	*D*_x_ = 1.479 Mg m^−^^3^
Monoclinic, *P*2_1_/*c*	Melting point = 486.4–483.1 K
Hall symbol: -P 2ybc	Cu *K*α radiation, λ = 1.54187 Å
*a* = 8.57511 (16) Å	Cell parameters from 22820 reflections
*b* = 14.0424 (3) Å	θ = 3.1–68.2°
*c* = 14.0842 (3) Å	µ = 2.31 mm^−^^1^
β = 100.206 (1)°	*T* = 193 K
*V* = 1669.12 (5) Å^3^	Platelet, yellow
*Z* = 4	0.60 × 0.40 × 0.30 mm

### Data collection

**Table:**

Rigaku R-AXIS RAPID diffractometer	3058 independent reflections
Radiation source: fine-focus sealed tube	2793 reflections with *I* > 2σ(*I*)
graphite	*R*_int_ = 0.028
Detector resolution: 10.00 pixels mm^-1^	θ_max_ = 68.2°, θ_min_ = 4.5°
ω scans	*h* = −10→10
Absorption correction: numerical (*NUMABS*; Higashi, 1999)	*k* = −16→16
*T*_min_ = 0.294, *T*_max_ = 0.544	*l* = −16→16
29408 measured reflections	

### Refinement

**Table:**

Refinement on *F*^2^	Secondary atom site location: difference Fourier map
Least-squares matrix: full	Hydrogen site location: inferred from neighbouring sites
*R*[*F*^2^ > 2σ(*F*^2^)] = 0.033	H-atom parameters constrained
*wR*(*F*^2^) = 0.093	*w* = 1/[σ^2^(*F*_o_^2^) + (0.0492*P*)^2^ + 0.5094*P*] where *P* = (*F*_o_^2^ + 2*F*_c_^2^)/3
*S* = 1.03	(Δ/σ)_max_ = 0.001
3058 reflections	Δρ_max_ = 0.21 e Å^−^^3^
238 parameters	Δρ_min_ = −0.19 e Å^−^^3^
0 restraints	Extinction correction: *SHELXL97* (Sheldrick, 2008), Fc^*^=kFc[1+0.001xFc^2^λ^3^/sin(2θ)]^-1/4^
Primary atom site location: structure-invariant direct methods	Extinction coefficient: 0.0049 (3)

### Special details

**Table:**

Geometry. All e.s.d.'s (except the e.s.d. in the dihedral angle between two l.s. planes) are estimated using the full covariance matrix. The cell e.s.d.'s are taken into account individually in the estimation of e.s.d.'s in distances, angles and torsion angles; correlations between e.s.d.'s in cell parameters are only used when they are defined by crystal symmetry. An approximate (isotropic) treatment of cell e.s.d.'s is used for estimating e.s.d.'s involving l.s. planes.
Refinement. Refinement of *F*^2^ against ALL reflections. The weighted *R*-factor *wR* and goodness of fit *S* are based on *F*^2^, conventional *R*-factors *R* are based on *F*, with *F* set to zero for negative *F*^2^. The threshold expression of *F*^2^ > σ(*F*^2^) is used only for calculating *R*-factors(gt) *etc*. and is not relevant to the choice of reflections for refinement. *R*-factors based on *F*^2^ are statistically about twice as large as those based on *F*, and *R*- factors based on ALL data will be even larger.

### Fractional atomic coordinates and isotropic or equivalent isotropic displacement parameters (Å^2^)

**Table:**

	*x*	*y*	*z*	*U*_iso_*/*U*_eq_	
Cl1	0.16621 (6)	1.13841 (3)	0.55672 (4)	0.06727 (18)	
O1	0.57537 (12)	0.75406 (7)	0.62980 (7)	0.0434 (3)	
O2	−0.00458 (14)	0.47410 (8)	0.31568 (8)	0.0511 (3)	
O3	0.41091 (13)	0.73567 (8)	0.38109 (7)	0.0468 (3)	
O4	0.06202 (13)	0.71291 (7)	0.40079 (8)	0.0485 (3)	
O5	0.11487 (19)	0.64395 (9)	0.27365 (8)	0.0676 (4)	
N1	0.11528 (15)	0.64617 (8)	0.35979 (9)	0.0412 (3)	
C1	0.38260 (16)	0.66673 (10)	0.52900 (9)	0.0338 (3)	
C2	0.49357 (17)	0.67065 (10)	0.61336 (10)	0.0360 (3)	
C3	0.52689 (18)	0.59082 (11)	0.67462 (10)	0.0405 (3)	
H3	0.5989	0.5956	0.7322	0.049*	
C4	0.45238 (18)	0.50699 (11)	0.64837 (11)	0.0415 (3)	
H4	0.4776	0.4538	0.6873	0.050*	
C5	0.26174 (18)	0.41072 (10)	0.53798 (11)	0.0408 (3)	
H5	0.2885	0.3585	0.5781	0.049*	
C6	0.15099 (18)	0.39956 (10)	0.45694 (11)	0.0410 (3)	
H6	0.1042	0.3404	0.4419	0.049*	
C7	0.10756 (17)	0.47805 (10)	0.39587 (10)	0.0377 (3)	
C8	0.17971 (16)	0.56535 (9)	0.42014 (9)	0.0347 (3)	
C9	0.29884 (16)	0.57978 (9)	0.50291 (9)	0.0329 (3)	
C10	0.33774 (17)	0.49855 (10)	0.56356 (10)	0.0366 (3)	
C11	0.32856 (16)	0.84584 (9)	0.48861 (10)	0.0350 (3)	
C12	0.25859 (19)	0.86117 (10)	0.56900 (11)	0.0427 (3)	
H12	0.2450	0.8103	0.6090	0.051*	
C13	0.2088 (2)	0.95114 (11)	0.59030 (12)	0.0470 (4)	
H13	0.1618	0.9612	0.6441	0.056*	
C14	0.23033 (19)	1.02564 (10)	0.53021 (12)	0.0457 (4)	
C15	0.3010 (2)	1.01289 (11)	0.45082 (12)	0.0501 (4)	
H15	0.3156	1.0642	0.4116	0.060*	
C16	0.35004 (18)	0.92290 (11)	0.43016 (11)	0.0423 (3)	
H16	0.3980	0.9136	0.3766	0.051*	
C17	0.37585 (16)	0.74943 (10)	0.46024 (10)	0.0350 (3)	
C18	0.6731 (2)	0.76752 (13)	0.72157 (11)	0.0501 (4)	
H18A	0.7157	0.8309	0.7256	0.060*	
H18B	0.7584	0.7223	0.7296	0.060*	
H18C	0.6113	0.7584	0.7714	0.060*	
C19	−0.0836 (2)	0.38502 (13)	0.29095 (13)	0.0560 (4)	
H19A	−0.1582	0.3921	0.2319	0.067*	
H19B	−0.1386	0.3661	0.3416	0.067*	
H19C	−0.0069	0.3373	0.2827	0.067*	

### Atomic displacement parameters (Å^2^)

**Table:**

	*U*^11^	*U*^22^	*U*^33^	*U*^12^	*U*^13^	*U*^23^
Cl1	0.0844 (3)	0.0300 (2)	0.0792 (3)	0.01301 (19)	−0.0079 (2)	−0.01089 (18)
O1	0.0501 (6)	0.0388 (6)	0.0398 (5)	−0.0041 (4)	0.0037 (4)	0.0022 (4)
O2	0.0594 (7)	0.0403 (6)	0.0494 (6)	−0.0012 (5)	−0.0019 (5)	−0.0042 (5)
O3	0.0609 (7)	0.0426 (6)	0.0418 (6)	0.0080 (5)	0.0226 (5)	0.0057 (4)
O4	0.0506 (6)	0.0319 (5)	0.0630 (7)	0.0123 (4)	0.0101 (5)	−0.0006 (5)
O5	0.1160 (12)	0.0474 (7)	0.0355 (6)	0.0052 (7)	0.0027 (6)	0.0034 (5)
N1	0.0494 (7)	0.0325 (6)	0.0400 (7)	0.0050 (5)	0.0031 (5)	0.0014 (5)
C1	0.0396 (7)	0.0292 (7)	0.0349 (7)	0.0073 (5)	0.0134 (5)	0.0021 (5)
C2	0.0401 (7)	0.0335 (7)	0.0366 (7)	0.0037 (6)	0.0128 (6)	0.0006 (5)
C3	0.0434 (8)	0.0412 (8)	0.0368 (7)	0.0069 (6)	0.0072 (6)	0.0046 (6)
C4	0.0472 (8)	0.0360 (8)	0.0421 (8)	0.0097 (6)	0.0106 (6)	0.0102 (6)
C5	0.0505 (8)	0.0282 (7)	0.0463 (8)	0.0070 (6)	0.0157 (7)	0.0067 (6)
C6	0.0488 (8)	0.0283 (7)	0.0488 (8)	0.0015 (6)	0.0164 (7)	−0.0014 (6)
C7	0.0415 (8)	0.0342 (7)	0.0393 (7)	0.0055 (6)	0.0126 (6)	−0.0030 (6)
C8	0.0420 (7)	0.0287 (7)	0.0356 (7)	0.0086 (5)	0.0128 (6)	0.0017 (5)
C9	0.0387 (7)	0.0289 (7)	0.0341 (7)	0.0079 (5)	0.0144 (5)	0.0017 (5)
C10	0.0427 (8)	0.0313 (7)	0.0382 (7)	0.0086 (6)	0.0140 (6)	0.0047 (5)
C11	0.0370 (7)	0.0296 (7)	0.0375 (7)	0.0001 (5)	0.0046 (5)	0.0030 (5)
C12	0.0536 (9)	0.0304 (7)	0.0467 (8)	0.0022 (6)	0.0154 (7)	0.0029 (6)
C13	0.0555 (9)	0.0356 (8)	0.0515 (9)	0.0040 (7)	0.0138 (7)	−0.0053 (7)
C14	0.0490 (9)	0.0272 (7)	0.0552 (9)	0.0027 (6)	−0.0061 (7)	−0.0038 (6)
C15	0.0652 (10)	0.0301 (8)	0.0511 (9)	−0.0034 (7)	−0.0002 (8)	0.0095 (6)
C16	0.0498 (8)	0.0359 (8)	0.0406 (7)	−0.0023 (6)	0.0063 (6)	0.0056 (6)
C17	0.0375 (7)	0.0330 (7)	0.0360 (7)	0.0013 (6)	0.0102 (6)	0.0028 (5)
C18	0.0544 (9)	0.0530 (9)	0.0413 (8)	−0.0073 (8)	0.0043 (7)	−0.0016 (7)
C19	0.0566 (10)	0.0471 (9)	0.0616 (10)	−0.0032 (8)	0.0026 (8)	−0.0134 (8)

### Geometric parameters (Å, °)

**Table:**

Cl1—C14	1.7384 (15)	C6—H6	0.9300
O1—C2	1.3639 (17)	C7—C8	1.388 (2)
O1—C18	1.4230 (18)	C8—C9	1.422 (2)
O2—C7	1.3485 (18)	C9—C10	1.4287 (18)
O2—C19	1.436 (2)	C11—C12	1.389 (2)
O3—C17	1.2202 (16)	C11—C16	1.3914 (19)
O4—N1	1.2303 (15)	C11—C17	1.4882 (19)
O5—N1	1.2130 (16)	C12—C13	1.383 (2)
N1—C8	1.4661 (17)	C12—H12	0.9300
C1—C2	1.385 (2)	C13—C14	1.378 (2)
C1—C9	1.4311 (19)	C13—H13	0.9300
C1—C17	1.5066 (18)	C14—C15	1.375 (2)
C2—C3	1.413 (2)	C15—C16	1.379 (2)
C3—C4	1.359 (2)	C15—H15	0.9300
C3—H3	0.9300	C16—H16	0.9300
C4—C10	1.411 (2)	C18—H18A	0.9600
C4—H4	0.9300	C18—H18B	0.9600
C5—C6	1.358 (2)	C18—H18C	0.9600
C5—C10	1.412 (2)	C19—H19A	0.9600
C5—H5	0.9300	C19—H19B	0.9600
C6—C7	1.407 (2)	C19—H19C	0.9600
			
C2—O1—C18	118.08 (11)	C4—C10—C9	119.53 (13)
C7—O2—C19	118.35 (12)	C12—C11—C16	118.90 (13)
O5—N1—O4	123.61 (12)	C12—C11—C17	122.51 (12)
O5—N1—C8	119.60 (12)	C16—C11—C17	118.54 (12)
O4—N1—C8	116.79 (11)	C13—C12—C11	120.84 (14)
C2—C1—C9	119.42 (12)	C13—C12—H12	119.6
C2—C1—C17	117.58 (13)	C11—C12—H12	119.6
C9—C1—C17	122.15 (12)	C14—C13—C12	118.70 (15)
O1—C2—C1	115.73 (12)	C14—C13—H13	120.7
O1—C2—C3	122.38 (13)	C12—C13—H13	120.7
C1—C2—C3	121.75 (14)	C15—C14—C13	121.78 (14)
C4—C3—C2	119.12 (14)	C15—C14—Cl1	119.68 (12)
C4—C3—H3	120.4	C13—C14—Cl1	118.54 (13)
C2—C3—H3	120.4	C14—C15—C16	119.06 (14)
C3—C4—C10	121.77 (13)	C14—C15—H15	120.5
C3—C4—H4	119.1	C16—C15—H15	120.5
C10—C4—H4	119.1	C15—C16—C11	120.70 (14)
C6—C5—C10	122.53 (13)	C15—C16—H16	119.6
C6—C5—H5	118.7	C11—C16—H16	119.6
C10—C5—H5	118.7	O3—C17—C11	120.81 (12)
C5—C6—C7	119.55 (14)	O3—C17—C1	118.53 (12)
C5—C6—H6	120.2	C11—C17—C1	120.66 (11)
C7—C6—H6	120.2	O1—C18—H18A	109.5
O2—C7—C8	117.58 (12)	O1—C18—H18B	109.5
O2—C7—C6	123.50 (13)	H18A—C18—H18B	109.5
C8—C7—C6	118.88 (14)	O1—C18—H18C	109.5
C7—C8—C9	123.49 (12)	H18A—C18—H18C	109.5
C7—C8—N1	115.79 (12)	H18B—C18—H18C	109.5
C9—C8—N1	120.47 (12)	O2—C19—H19A	109.5
C8—C9—C10	115.73 (12)	O2—C19—H19B	109.5
C8—C9—C1	125.95 (12)	H19A—C19—H19B	109.5
C10—C9—C1	118.31 (13)	O2—C19—H19C	109.5
C5—C10—C4	120.69 (13)	H19A—C19—H19C	109.5
C5—C10—C9	119.78 (13)	H19B—C19—H19C	109.5
			
C18—O1—C2—C1	170.71 (12)	C2—C1—C9—C10	−2.42 (18)
C18—O1—C2—C3	−13.54 (19)	C17—C1—C9—C10	166.79 (12)
C9—C1—C2—O1	175.92 (11)	C6—C5—C10—C4	−179.90 (13)
C17—C1—C2—O1	6.22 (17)	C6—C5—C10—C9	−0.1 (2)
C9—C1—C2—C3	0.14 (19)	C3—C4—C10—C5	−179.85 (13)
C17—C1—C2—C3	−169.56 (12)	C3—C4—C10—C9	0.4 (2)
O1—C2—C3—C4	−173.05 (12)	C8—C9—C10—C5	1.55 (18)
C1—C2—C3—C4	2.4 (2)	C1—C9—C10—C5	−177.58 (12)
C2—C3—C4—C10	−2.7 (2)	C8—C9—C10—C4	−178.68 (12)
C10—C5—C6—C7	−0.7 (2)	C1—C9—C10—C4	2.19 (19)
C19—O2—C7—C8	−178.28 (13)	C16—C11—C12—C13	0.9 (2)
C19—O2—C7—C6	−0.3 (2)	C17—C11—C12—C13	−176.66 (14)
C5—C6—C7—O2	−177.91 (13)	C11—C12—C13—C14	−0.1 (2)
C5—C6—C7—C8	0.1 (2)	C12—C13—C14—C15	−0.7 (2)
O2—C7—C8—C9	179.59 (12)	C12—C13—C14—Cl1	179.50 (13)
C6—C7—C8—C9	1.5 (2)	C13—C14—C15—C16	0.8 (2)
O2—C7—C8—N1	5.20 (18)	Cl1—C14—C15—C16	−179.43 (12)
C6—C7—C8—N1	−172.92 (12)	C14—C15—C16—C11	0.0 (2)
O5—N1—C8—C7	−58.76 (19)	C12—C11—C16—C15	−0.8 (2)
O4—N1—C8—C7	120.32 (14)	C17—C11—C16—C15	176.83 (14)
O5—N1—C8—C9	126.67 (15)	C12—C11—C17—O3	167.06 (14)
O4—N1—C8—C9	−54.26 (18)	C16—C11—C17—O3	−10.5 (2)
C7—C8—C9—C10	−2.25 (19)	C12—C11—C17—C1	−13.4 (2)
N1—C8—C9—C10	171.89 (12)	C16—C11—C17—C1	169.08 (13)
C7—C8—C9—C1	176.80 (12)	C2—C1—C17—O3	114.72 (15)
N1—C8—C9—C1	−9.1 (2)	C9—C1—C17—O3	−54.68 (18)
C2—C1—C9—C8	178.55 (12)	C2—C1—C17—C11	−64.85 (17)
C17—C1—C9—C8	−12.2 (2)	C9—C1—C17—C11	125.75 (14)

### Hydrogen-bond geometry (Å, °)

**Table:**

Cg1 is the centroid of the C1–C4/C9/C10 ring.

**Table:**

*D*—H···*A*	*D*—H	H···*A*	*D*···*A*	*D*—H···*A*
C13—H13···O5^i^	0.93	2.44	3.137 (2)	132
C18—H18C···O3^i^	0.96	2.51	3.449 (2)	167
C19—H19B···Cg1^ii^	0.96	2.81	3.5845 (19)	139

Symmetry codes: (i) *x*, −*y*+3/2, *z*+1/2; (ii) −*x*+2, −*y*+1, −*z*+1.
